# Low-Level Laser Therapy Reduces Lung Inflammation in an Experimental Model of Chronic Obstructive Pulmonary Disease Involving P2X7 Receptor

**DOI:** 10.1155/2018/6798238

**Published:** 2018-03-04

**Authors:** Gabriel da Cunha Moraes, Luana Beatriz Vitoretti, Auriléia Aparecida de Brito, Cintia Estefano Alves, Nicole Cristine Rigonato de Oliveira, Alana dos Santos Dias, Yves Silva Teles Matos, Manoel Carneiro Oliveira-Junior, Luis Vicente Franco Oliveira, Renata Kelly da Palma, Larissa Carbonera Candeo, Adriana Lino-dos-Santos-Franco, Anna Carolina Ratto Tempestine Horliana, João Antonio Gimenes Júnior, Flavio Aimbire, Rodolfo Paula Vieira, Ana Paula Ligeiro-de-Oliveira

**Affiliations:** ^1^Post Graduate Program in Biophotonics Applied to Health Sciences, University Nove de Julho (UNINOVE), Sao Paulo, SP, Brazil; ^2^Brazilian Institute of Teaching and Research in Pulmonary and Exercise Immunology (IBEPIPE), São José dos Campos, SP, Brazil; ^3^Experimental Cardiorespiratory Physiology Laboratory, Master's Degree and PhD Program in Rehabilitation Sciences, University Nove de Julho (UNINOVE), Sao Paulo, SP, Brazil; ^4^Division of Trauma, Surgical Critical Care, Burns, and Acute Care Surgery, Department of Surgery, University of California San Diego (UCSD) Health Sciences, San Diego, CA, USA; ^5^Institute of Science and Technology, Federal University of São Paulo (UNIFESP), Sao José dos Campos, SP, Brazil; ^6^Post-graduation Program in Bioengineering, Universidade Brasil, São Paulo, SP, Brazil; ^7^Post-graduation Program in Sciences of Human Movement and Rehabilitation, Federal University of São Paulo (UNIFESP), Santos, SP, Brazil

## Abstract

Chronic obstructive pulmonary disease (COPD) is a progressive disease characterized by irreversible airflow limitation, airway inflammation and remodeling, and enlargement of alveolar spaces. COPD is in the top five leading causes of deaths worldwide and presents a high economic cost. However, there are some preventive measures to lower the risk of developing COPD. Low-level laser therapy (LLLT) is a new effective therapy, with very low cost and no side effects. So, our objective was to investigate if LLLT reduces pulmonary alterations in an experimental model of COPD. C57BL/6 mice were submitted to cigarette smoke for 75 days (2x/day). After 60 days to smoke exposure, the treated group was submitted to LLLT (diode laser, 660 nm, 30 mW, and 3 J/cm^2^) for 15 days and euthanized for morphologic and functional analysis of the lungs. Our results showed that LLLT significantly reduced the number of inflammatory cells and the proinflammatory cytokine secretion such as IL-1*β*, IL-6, and TNF-*α* in bronchoalveolar lavage fluid (BALF). We also observed that LLLT decreased collagen deposition as well as the expression of purinergic P2X7 receptor. On the other hand, LLLT increased the IL-10 release. Thus, LLLT can be pointed as a promising therapeutic approach for lung inflammatory diseases as COPD.

## 1. Introduction

Chronic obstructive pulmonary disease (COPD) is a global health problem and has been predicted to become the third cause of death in the world by 2020 [1]. Cigarette smoking is currently the major cause of COPD, but recent studies have described a significant prevalence of COPD among never-smokers. The estimated annual costs of COPD in the USA are $50 billion, and most of these costs are related to exacerbations requiring hospitalization [1, 2]. COPD is characterized by airflow limitation that is not fully reversible and is usually progressive and associated with an abnormal inflammatory response of lungs [3].

Low-level laser therapy (LLLT) has been used clinically since 1981 in the treatment of patients with inflammatory pathologies [4]. It is a relatively new and promising approach, with very low cost, no invasiveness, and no side effects. The scientific literature has reported anti-inflammatory effects of LLLT for treating musculoskeletal aches and pains, wound healing, and chronic and acute inflammation [5]. Furthermore, a growing number of clinical studies are demonstrating the efficacy and safety of LLLT for different pulmonary diseases, as asthma and COPD [6, 7]. For instance, some studies also have demonstrated that the application of LLLT for the treatment of patients with chronic obstructive bronchitis accelerates the elimination of clinical symptoms, increases its efficiency, promotes drainage function of the bronchi, facilitates standardization of the immune status of the patient, and contributes to the optimization of lipid peroxidation [6, 7].

Extracellular ATP has recently gained attention as a danger signal and important mediator of inflammation via the activation of purinergic receptors of the P2X (P2X1–P2X7) and P2Y type (P2Y1, P2Y2, P2Y4, P2Y6, and P2Y11–P2Y14). During hypoxia, trauma, and infection or inflammation, extracellular ATP levels can markedly rise, either by active or by passive release from various cell types, such as lung epithelial cells and inflammatory cells [8, 9]. ATP neutralization or the inhibition of purinergic receptors can prevent smoke-induced lung inflammation by reducing neutrophil and macrophage infiltration and the release of proinflammatory cytokines, such as IL-1*β*, MIP-2, CXCL1/KC, IFN-*γ*, and IL-6 in bronchoalveolar lavage fluid (BALF) [10].

In this study, we provide evidence that LLLT is effective in reducing lung inflammation, as well as the production and deposition of collagen in the lung parenchyma of COPD animals. Moreover, the data suggest that ATP is linked to the pathogenesis of cigarette smoke-induced COPD, since LLLT decreased the expression of P2X7 receptor.

## 2. Material and Methods

### 2.1. Animals

Female C57BL/6 mice (weight range 19–22 g, 6–8 weeks old) were obtained from the University Nove de Julho and maintained in a 12 h light/dark cycle (lights on at 7:00 a.m. daily), with a controlled temperature at 21 ± 0.3°C and relative humidity at 50 ± 4%, with free access to rodent food and water. All experiments carried out in this study were approved by the Animal Care Committee University Nove de Julho.

### 2.2. Experimental Model of Cigarette Smoke-Induced Emphysema

Whole-body cigarette smoke exposure was performed according to an adapted protocol as described previously by Peron et al. [11]. Briefly, mice were exposed to cigarette smoke of 7 commercially available cigarettes (each one containing 13 mg of tar, 1.10 mg of nicotine, and 10 mg of carbon monoxide) during 75 days, twice a day, 30 minutes each session. Animals exposed to ambient air served as a control. All experiments were carried out on day 76.

### 2.3. Low-Level Laser Treatment Protocol

A diode laser (power 30 mW, energy density of 3 J/cm^2^ at 660 nm of wavelength) was used. LLLT was performed twice a day, starting from day 60 up to day 75 of the cigarette smoke exposure protocol. One hour after each session of cigarette exposure, the diode laser was applied directly on the skin, over the trachea and the lung lobes, for 30 seconds each point, using a small spot size (0.785 cm^2^). The animals were divided into three experimental groups: basal (mice exposed to ambient air), COPD (animals exposed to cigarette smoke without LLLT), and COPD + LLLT.

### 2.4. Bronchoalveolar Lavage Fluid

Mice were anaesthetized and submitted to tracheotomy. Then, the lungs were flushed 3 times with 0.5 ml of PBS each time. The recovery BALF was centrifuged at 450*g*, at 4°C, for 15 minutes. The supernatant fraction was stored at −80°C for further cytokine analysis, and the cell pellet was resuspended in 1.0 ml of PBS. Total cells were counted using a hematocytometer (Neubauer chamber). Differential cell analyses were performed using a cytocentrifuge (Cytospin) preparation (200 *μ*l of BALF was centrifuged at 300*g* for 10 minutes). Cells were stained with the May-Grünwald-Giemsa method, and 300 cells were counted according to their morphological characteristics [12, 13].

### 2.5. Pulmonary Cytokine Levels

Levels of IL-1*β*, IL-6, CINC-1/KC, IL-17, TNF-*α*, and IL-10 were assessed in BALF supernatants using ELISA kits (R&D Systems, Minneapolis), according to the manufacturer's instruction.

### 2.6. Peribronchial Inflammation Analysis

The space between the airway basement membrane and adventitia was determined using Image Pro Plus software as the target field. The number and type of cells (mononuclear and polymorphonuclear cells) were assessed in this specific area. The results were expressed as number of cells per square millimeter [12].

### 2.7. Airway Remodeling Assessment by Collagen Deposition

The lungs were kept in formaldehyde 10% at 20 cmH_2_O pressure for 24 hours before inclusion in paraffin. 5 *μ*m sections of the lungs were stained with Sirius Red. The area between the airway basement membrane and the airway adventitia was considered the target field, and positive stained areas (red staining) were evaluated [12, 14].

### 2.8. Determination of Emphysema Levels by Alveolar Enlargement

To measure the alveolar enlargement, sections of lung tissue were stained with hematoxylin and eosin, and airspace enlargement was assessed by the mean linear intercept (Lm) in twenty selected fields per slide, at 200x magnification [14]. Destruction of alveolar septa was evaluated by a point-counting technique in twenty fields randomly throughout the lung parenchyma (excluding fields presenting airways and pulmonary vessels) [14].

### 2.9. Western Blotting for P2X7 Receptor

Lung samples were homogenized in RIPA lysis buffer (Santa Cruz, CA) and centrifuged at 13300 rpm at 4°C for 15 minutes. Protein was measured by BCA (BCA Protein Assay kit, Thermo Scientific, USA). 50 *μ*g of proteins (which was composed of a pool of the same amount of proteins of 8 mice from each group) was loaded in NuPAGE 4–12% Bis-Tri gel (Invitrogen, USA) and transferred to a nitrocellulose membrane. Primary antibody anti-P2X7 receptor (sc-15200, Santa Cruz, USA) was visualized using a horseradish-conjugated secondary antibody and enhanced by chemiluminescence (Pierce, Thermo Scientific, USA). An additional probe with *β*-actin for control of the amount of proteins was applied using a monoclonal anti-actin antibody clone C4 (sc-130656, Santa Cruz, USA). The densitometric analysis of the expression of the bands was performed using the ImageJ from NIH.

### 2.10. Lung Mechanics Analysis

The end-inspiratory occlusion after the constant-flow inflation method was used to assess pulmonary mechanics. The first measurements were done in a close-chest model, followed by additional measurements in an open-chest model [13, 14]. Then, tracheal pressure was measured, which reflects transpulmonary pressure (PL). The initial fast drop in PL (ΔP1) occurs immediately after end-inspiratory occlusion from the preocclusion corresponding to the inflection point (*P*_i_), followed by a slow pressure decay (ΔP2) when the plateau is reached, corresponding to the elastic recoil pressure of the lung (*P*_el_). ΔP1 significates the dissipated pressure against pulmonary resistance, while ΔP2 reflects viscoelastic properties (stress relaxation) [13, 14]. Total pressure drop (Δ*P*_tot_) corresponds to ΔP1 + ΔP2. Dynamic elastance (*E*_dyn_) and static elastance (*E*_st_) was obtained by dividing *P*_el_ and *P*_i_, respectively, by lung volume (VT). The difference *E*_dyn_ − *E*_st_ corresponds to Δ*E*. Data analysis was performed with ANADAT software (RHT-InfoData, Montreal, Canada) [13, 14].

### 2.11. Statistical Analysis

The normality of the data was tested by the Shapiro-Wilk test, and the data was analyzed by one-way analysis of variance (ANOVA) followed by the Newman-Keuls posttest. Analyses were performed using GraphPad Prism software 5.0 (GraphPad Software Inc.). Data are expressed as mean ± SD. *P* values less than 0.05 were considered statistically significant.

## 3. Results

### 3.1. LLLT Reduces Leukocytes in BALF and in Lung Tissue

**Figure 1:**
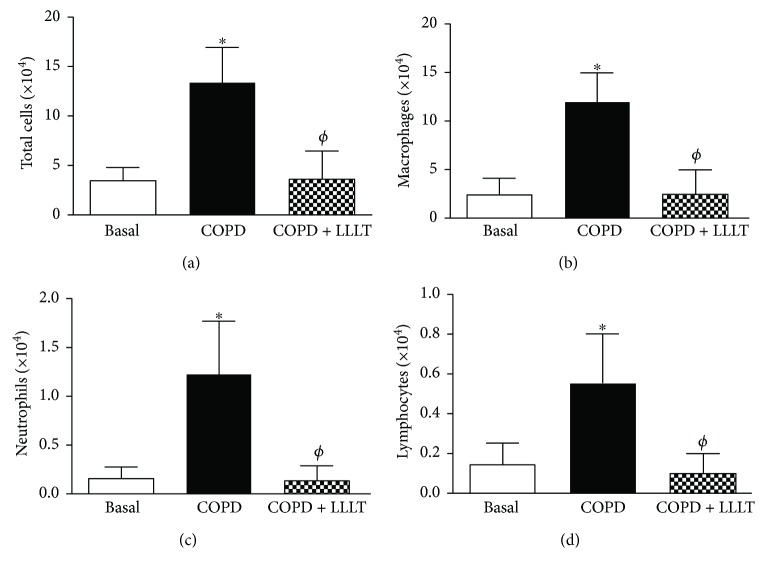
Treatment with LLL reduces cellular infiltration in BALF of COPD animals. Quantification of total cells (a), macrophages (b), neutrophils (c), and lymphocytes (d) in bronchoalveolar lavage fluid. Data are expressed as mean ± SD of three independent experiments. *n* = 5–8 animals per group. ^∗^*P* < 0.05 in relation to the basal group; ^*ϕ*^*P* < 0.05 in relation to the COPD group.

Data obtained showed that cigarette smoke exposure in the COPD group promoted a significant increase in total leukocyte influx in BALF (Figure 1(a)), as well as in the number of macrophages, neutrophils, and lymphocytes (Figures 1(b)–1(d)), which was reduced by LLLT.

**Figure 2:**
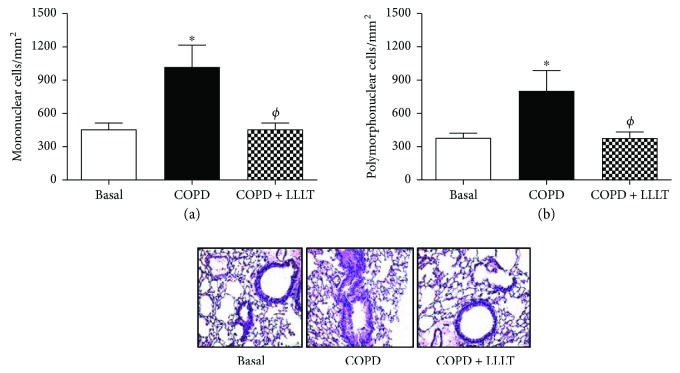
Treatment with LLL reduces mononuclear and polymorphonuclear cells in peribronchial space of COPD animals. Quantification of mononuclear (a) and polymorphonuclear (b) cells in peribronchial space in lung parenchyma. Data are expressed as mean ± SD of three independent experiments. *n* = 5–8 animals per group. ^∗^*P* < 0.05 in relation to the basal group; ^*ϕ*^*P* < 0.05 in relation to the COPD group.

The same was observed in lung parenchyma, where the number of mononuclear and polymorphonuclear cells decreased after LLLT (Figures 2(a) and 2(b)).

### 3.2. LLLT Reduces Inflammatory Cytokines in the BALF

**Figure 3:**
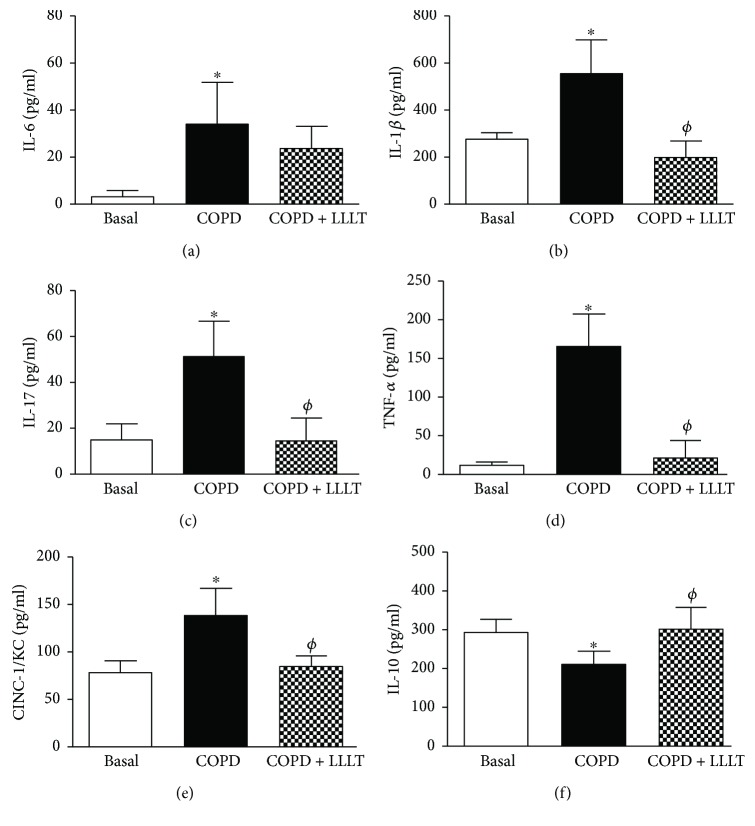
Treatment with LLL reduces proinflammatory cytokine secretion and increases the levels of IL-10 in the BALF of COPD animals. Quantification of IL-6 (a), IL-1*β* (b), IL-17 (c), TNF-*α* (d), KC (e), and IL-10 (f) in bronchoalveolar lavage fluid supernatants. Data are expressed as mean ± SD of three independent experiments. *n* = 5–8 animals per group. ^∗^*P* < 0.05 in relation to the basal group; ^*ϕ*^*P* < 0.05 in relation to the COPD group.

The levels of the proinflammatory cytokines IL-6, IL-1*β*, IL-17, TNF-*α*, and CINC-1/KC in BALF supernatants increased in the COPD group and were significantly reduced by LLLT. On the other hand, the LLLT was capable to reestablish the levels of the anti-inflammatory cytokine IL-10 that were reduced by the cigarette smoke exposure in the COPD group (Figure 3).

### 3.3. LLLT Reduces Collagen Deposition and Alveolar Enlargement in the Lungs of COPD Animals

**Figure 4:**
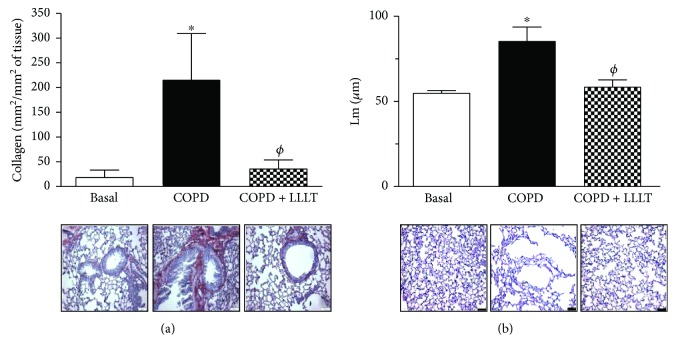
Treatment with LLL reduces collagen deposition and alveolar enlargement in the lungs of COPD animals. The sections were stained with Sirius Red for collagen detection (a) and hematoxylin/eosin for alveolar enlargement (b). Data are expressed as mean ± SD. ^∗^*P* < 0.05 in relation to the basal group; ^*ϕ*^*P* < 0.05 in relation to the COPD group.

We next decided to verify some possible effects of LLLT on collagen deposition and on the enlargement of lung parenchyma, both considered important markers for COPD. Our data demonstrated that the amount of collagen in the COPD group is higher than that in the control group. After this, LLLT significantly decreased the collagen in the airways of all animals under therapeutic protocol (Figure 4(a)). We also observed that the LLLT significantly decreases the alveolar enlargement when compared to the COPD group, indicating reduced lung emphysema (Figure 4(b)).

### 3.4. LLLT Reduces the Expression of Purinergic P2X7 Receptor

**Figure 5:**
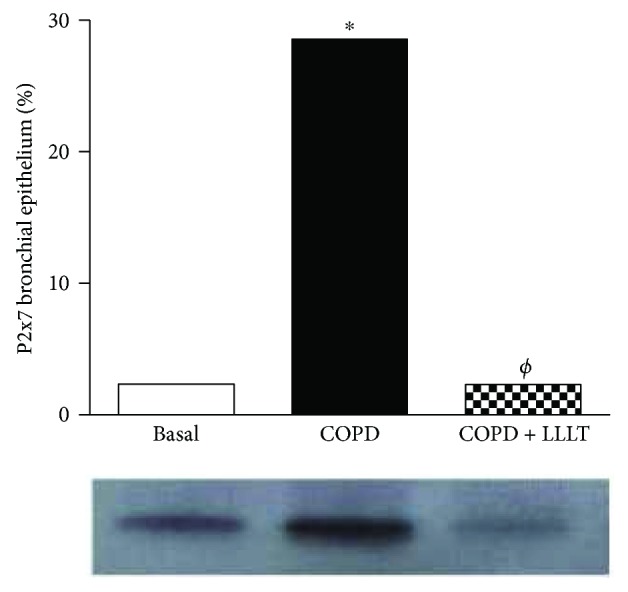
Treatment with LLL reduces P2X7 receptor expression in the lungs of COPD animals. Lung samples were homogenized to determine the expression of the P2X7 receptor by the Western blotting technique. ^∗^*P* < 0.05 in relation to the basal group; ^*ϕ*^*P* < 0.05 in relation to the COPD group.

We analyzed the expression of purinergic P2X7 receptor in lung tissue of mice after cigarette smoke exposure. The results showed that LLLT significantly reduced the increase in expression of the purinergic P2X7 receptor in smoke-induced COPD animals as shown in Figure 5.

### 3.5. LLLT Increases Lung Mechanics

**Figure 6:**
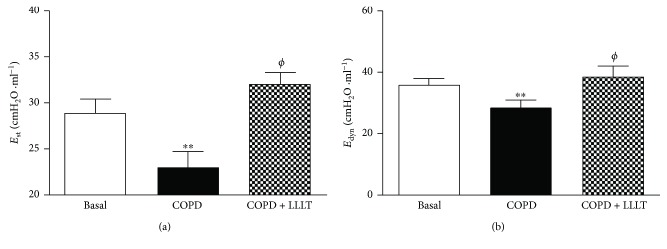
Effect of treatment with LLL on static (a) and dynamic (b) elastance in the respiratory system. The animals were submitted to the anterior incision of the trachea, followed by cannulation of the same. Data are expressed as mean ± SD. *n* = 5–8 animals per group. ^∗∗^*P* < 0.01 in relation to the basal group; ^*ϕ*^*P* < 0.05 in relation to the COPD group.

**Figure 7:**
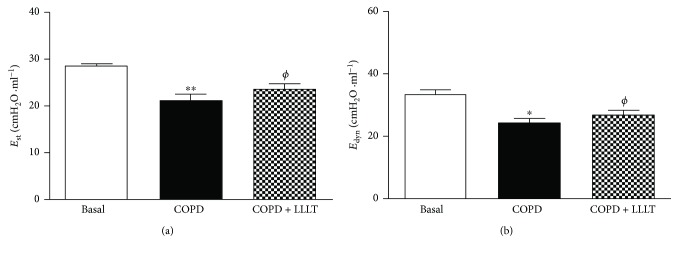
Effect of treatment with LLL on static (a) and dynamic (b) elastance in the lungs. The animals were submitted to the anterior incision of the trachea, followed by cannulation of the same. Data are expressed as mean ± SD. *n* = 5–8 animals per group. ^∗^*P* < 0.05 and ^∗∗^*P* < 0.01 in relation to the basal group; ^*ϕ*^*P* < 0.05 in relation to the COPD group.

As shown in Figures 6 and 7, the respiratory system and lung elastance values (*E*_st_ and *E*_dyn_) measured were reduced in the COPD group when compared to the basal group. On the other hand, we observed that the LLLT significantly increases these elastance values.

## 4. Discussion

The present study showed that the LLLT (660 nm) in an experimental model reduces the main COPD outcomes, such as lung emphysema, airway remodeling, and chronic bronchitis. In addition, the study showed that such effects were followed by reduced expression of P2X7 receptor, suggesting LLLT modulating purinergic signaling, a molecular pathway involved in the pathogenesis of COPD. In summary, LLLT reduced BAL cellularity, proinflammatory cytokine secretion, collagen deposition, alveolar enlargement, and the expression of the P2X7 receptor.

Despite cigarette smoking-associated diseases being the fourth leading cause of death in the United States of America [15], there is a clear need for new therapies that can prevent the induction and the progression of COPD [16]. In the physiopathology of COPD, macrophages and neutrophils contribute to the lung injury releasing several mediators, free radicals, proteases, cytokines, and chemokines [15]. Furthermore, the activation of neutrophils has been directly linked with COPD severity and mortality. In this way, our results showed that LLLT was effective to reduce the migration of these cells into the lungs, as well as to inhibit proinflammatory cytokine secretion, reinforcing the beneficial effects of LLL therapy. Furthermore, some studies have evaluated the lung inflammation with special emphasis on airway remodeling. The airway remodeling requires cellular proliferation, increased deposition of extracellular matrix proteins, thickening of the basement membrane, collagen production, and increased mucus secretion [16, 17]. In our study, we have observed that LLL-treated mice have displayed a significant reduction in collagen deposition and improved alveolar enlargement, which are key features of COPD. In this context, the laser therapy seems to contribute to the maintenance of integrity of lung structure. Such beneficial effects of LLLT observed in the present study were reinforced by functional measurements, as demonstrated by improved static and dynamic elastance, evaluated in both close- and open-chest fashion. These results are particularly important, since reduced static elastance and dynamic elastance observed in the present study resemble typical impairment of human COPD and are related to increased work of breathing, significantly accounting to COPD severity [18].

The main mechanisms responsible for the modulation of the inflammatory process triggered by LLLT involve increasing local microcirculation, promotion of angiogenesis, and immune cell activation leading to accelerated healing [19]. The scientific literature about the effects of LLLT in COPD is scarce; however, recently we have demonstrated that the LLLT (660 nm) alone and in association with human tubal-derived mesenchymal stromal cells reduced cigarette smoke-induced COPD [11]. Corroborating our findings, several other groups have already addressed the capacity of LLLT in modulating lung diseases. Miranda da Silva et al. [20], in a model of lung inflammation induced by formaldehyde exposure, demonstrated that LLLT (660 nm) reduced the number of leukocytes, mast cell degranulation, myeloperoxidase activity, microvascular lung permeability, and inflammatory cytokine release. In the same way, Silva et al. [21] demonstrated that LLLT (660 nm) reduced bronchial hyperresponsiveness, eosinophils and eotaxin, ICAM expression, and Th2 cytokine release in a model of experimental allergic lung inflammation. In another study, Oliveira et al. [22] showed, for the first time, the effects of LLLT (830 nm) reducing the acute pulmonary inflammation in a pulmonary and extra pulmonary model of LPS-induced ARDS in BALB/c mice.

Interestingly, recent reports have shown that LLLT also exerts its function by increasing intracellular AMPc, thus suppressing important inflammatory transcription factors as NF-*κ*B [23]. Moreover, studies showed that ATP was elevated in BALF of cigarette smoke-exposed mice [24] and of human cigarette smokers [25]. The P2X7 receptor when exposed to ATP induces a number of features associated with COPD pathogenesis, such as neutrophil chemotaxis, release of proinflammatory cytokines (i.e., IL-1*β*, IL-6, and IL-18), reactive oxygen species (ROS), and tissue-degrading enzymes from neutrophils and macrophages [25]. In fact, Lommatzsch et al. [25] showed that the P2X7 receptor is upregulated on BALF macrophages and blood neutrophils from patients with COPD. In the present study, we demonstrated for the first time that LLLT reduces smoke-induced P2X7 receptor expression in lung tissue, suggesting an involvement of this receptor as part of the mechanisms of LLLT reducing smoke-induced COPD in mice.

In summary, our results indicate that LLLT can be a promising therapy in COPD treatment, as demonstrated in an experimental model of cigarette smoke-induced COPD in mice.
